# Surface Passivation of Perovskite Solar Cells Toward Improved Efficiency and Stability

**DOI:** 10.1007/s40820-019-0282-0

**Published:** 2019-06-07

**Authors:** Zhiqi Li, Jiajun Dong, Chunyu Liu, Jiaxin Guo, Liang Shen, Wenbin Guo

**Affiliations:** 1 State Key Laboratory on Integrated Optoelectronics, College of Electronic Science and Engineering, Jilin University, 2699 Qianjin Street, Changchun, 130012 People’s Republic of China; 2 State Key Laboratory of Superhard Materials, Jilin University, 2699 Qianjin Street, Changchun, 130012 People’s Republic of China

**Keywords:** Perovskite solar cells, Surface passivation, Charge transport, Surface defect

## Abstract

The advancement of perovskite solar cells (PVSCs) technology toward commercialized promotion needs high efficiency and optimum stability. By introducing a small molecular material such as tetratetracontane (TTC, CH_3_(CH_2_)_42_CH_3_) at the fullerene (C_60_)/perovskite interface of planar p-i-n PVSCs, we significantly reduced the interfacial traps, thereby suppressing electron recombination and facilitating electron extraction. Consequently, an improved efficiency of 20.05% was achieved with a high fill factor of 79.4%, which is one of the best performances for small molecular-modified PVSCs. Moreover, the hydrophobic TTC successfully protects the perovskite film from water damage. As a result, we realized a better long-term stability that maintains 87% of the initial efficiency after continuous exposure for 200 h in air.

**Electronic supplementary material:**

The online version of this article (10.1007/s40820-019-0282-0) contains supplementary material, which is available to authorized users.

## Highlights

- The TTC layer was efficiently deposited at the grain boundary of the perovskite, which passivated the grain surface and grain boundary, thereby decreasing the interfacial recombination of the perovskite solar cells.
- The hydrophobic small molecule TTC on the perovskite films forms a water-resistant layer that protects the perovskite from water damage.

**Electronic supplementary material:**

The online version of this article (10.1007/s40820-019-0282-0) contains supplementary material, which is available to authorized users.

## Graphical abstract

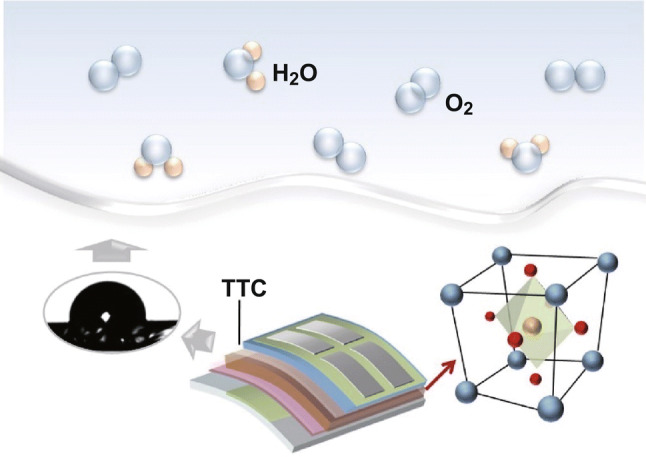

## Introduction

Organic lead halide perovskite materials such as methylammonium lead iodide (CH_3_NH_3_PbI_3_) have been revitalizing worldwide photovoltaic research due to the superb photoelectric properties [1–4]. More encouragingly, the hybrid perovskite solar cells (PVSCs) possess the potential to be highly scalable and manufactured by low-cost solution routes [5–7]. Today, the certificated efficiency of PVSCs has already reached 24.2%, rivaling their contemporary inorganic counterparts [8]. However, the PVSCs still suffer from instability, especially when exposed to moisture. Therefore, their sensitivity against moisture needs to be rationally addressed before they can be considered as commercially viable [9].

The decomposition of perovskite in humid ambience is mainly ascribed to the hydrolysis of CH_3_NH_3_PbI_3_. The combination of H_2_O and CH_3_NH_3_PbI_3_ forms PbI_2_ and CH_3_NH_3_I, and the latter further degrades into the HI and CH_3_NH_2_ [10]. PbI_2_ dissolves out of the perovskite, resulting in a porous structure that in turn accelerates H_2_O and O_2_ absorption, HI reacts with O_2_ to form I_2_ and H_2_O, which can further drive the degradation process and decrease device efficiency [11–13].Therefore, to overcome the moisture instabilities, numerous strategies have been proposed, such as additive promoted crystallization [14], mixed-dimensional perovskite preparation [15], modification of the electron/hole transport layer [16, 17], and device encapsulation [18]. Recently, many groups have succeeded in utilizing the interface engineering strategy to minimize the influence of moisture and improve the stability. Likewise, many interfacial materials have been investigated (Table S1), including metal oxides [19], polymers [20–23], and carbon-based materials [24]. These hydrophobic interfaces can not only substantially limit the permeation of atmospheric moisture but also enhance the device performance in terms of decreasing surface recombination, tuning band energy offsets, and optimizing interfacial contact [25]. Among these, small organic molecules have attracted interest for their easy synthesis, purification, and reproducible property [26, 27]. A slight chemical modification of the molecules’ structure enables further fine-tuning of the interfacial properties according to the research requirements [28]. Additionally, organic molecules can be deposited by solution processing and vacuum evaporation, making this a scalable technique in future [29]. Considering the above advantages, interface strategy of small molecule is essential to enhance the performance and stability of the PVSCs.

In this work, we developed an organic small molecule tetratetracontane (TTC, CH_3_(CH_2_)_42_CH_3_) as an interlayer for planar p-i-n PVSCs. Based on the device structure of ITO/poly(triarylamine)(PTAA):2,3,5,6-tetrafluoro-7,7,8,8-tetracyanoquinodimethane (F_4_-TCNQ)/CH_3_NH_3_PbI_3_/TTC/fullerene(C_60_)/BCP/Ag, we achieved a high power conversion efficiency (PCE) of 20.05% with a high fill factor (FF) of 79.4%, compared with that of 17.38% achieved by the control device. The TTC passivation layer reduces the defects at the perovskite surface, which suppresses electron recombination and facilitates electron extraction. Moreover, the hydrophobic TTC can function as a water-resistant layer and protect the device from water damage, leading to highly stable perovskite devices.

## Experimental Section

### Materials and Sample Preparation

PbI_2_, methylammonium iodide (MAI), and PTAA were purchased from Xi’an Polymer Light Technology Corp. *N*,*N*-Dimethylformamide (DMF) (99.8%), dimethyl sulfoxide (DMSO) (99.8%), and F_4_-TCNQ were received from Sigma-Aldrich. C_60_ and BCP were purchased from American Dye Source Inc. All materials mentioned above were used as received without further purification.

### PTAA Precursor Preparation

The PTAA solution was prepared by dissolving PTAA in toluene (Sigma-Aldrich) with a concentration of 2 mg mL^−1^ and stirred overnight.

### Perovskite Precursor Preparation

The perovskite precursor solution was prepared via mixing 462 mg of MAI, 159 mg of PbI_2_, and 78 μL of DMSO (a molar ratio of 1:1:1) powder in 600 μL of anhydrous DMF. The solution was stirred overnight at room temperature and filtered with 0.22 μm PVDF filters before device fabrication.

### Fabrication of Perovskite Solar Cells

The configuration of the fabricated devices was ITO/PTAA:F_4_TCNQ/CH_3_NH_3_PbI_3_/TTA/C_60_/BCP/Ag. ITO (15 O sq^−1^) glass substrates were cleaned sequentially with detergent, deionized water, acetone, and isopropanol followed by drying with N_2_ stream and UV–ozone treatment for 10 min. The PTAA:F_4_-TCNQ hole transport layers were formed by spin coating onto the cleaned ITO substrates at 4000 rpm for 30 s and annealed on the hot plate at 100 °C for 15 min in air. Then, the perovskite precursor solution was spin-coated onto the top of the prepared substrates at 4000 rpm for 30 s and annealed at 100 °C for 10 min. After the CH_3_NH_3_PbI_3_ film was formed and cooled to room temperature, TTA layers of different thicknesses were deposited by thermal evaporation on the top of the perovskite. Afterward, 25 nm C_60_ and 6 nm BCP were sequentially deposited by thermal evaporation under a vacuum of 5 × 10^−4^ Pa. Finally, a Ag electrode of 100 nm thickness was evaporated through a shadow mask. The device area was defined as 4 mm^2^ for each solar cell discussed in this work, and all of the above processes were executed completely in air at room temperature.

### Device Characterization

The device photocurrent was recorded using a Keithley 2400 Source Meter unit under AM1.5 illumination condition at an intensity of 100 mW cm^−2^ in air. The illumination intensity of the light source was accurately calibrated with a standard Si solar cell. The incident photon-to-electron conversion efficiency (IPCE) was measured using a Newport Oriel IPCE measurement kit. The light intensity was calibrated using a single-crystal Si photovoltaic cell. The scanning electron microscopy (SEM) images were taken on a ZEISS Sigma field-emission scanning electron microscopy (FE-SEM). The X-ray diffraction (XRD) patterns were recorded on a Rigaku Ultima IV diffractometer using Cu Kα radiation. The UV–Vis absorption spectra were measured using a UV-1700 spectrometer.

## Results and Discussion

**Fig. 1:**
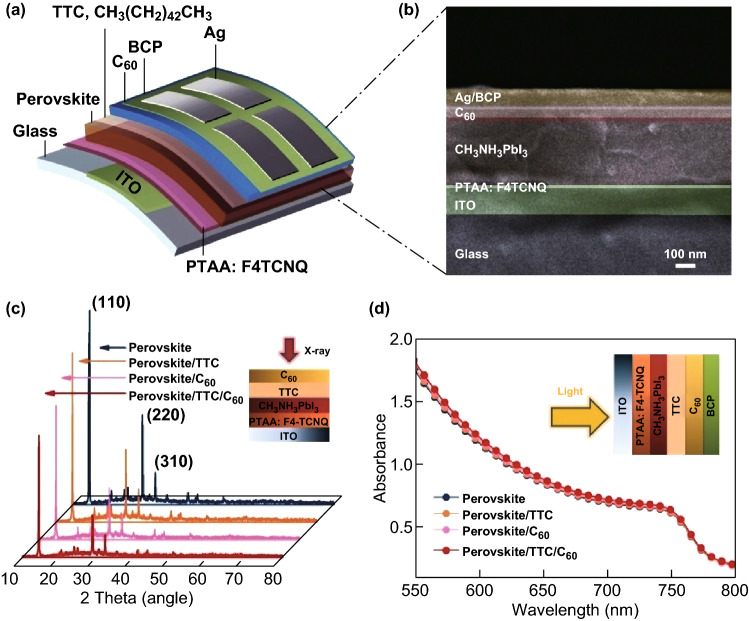
**a** Device structure used in this study. **b** Cross-sectional SEM image of the full device, including a TTC interlayer (scale bar: 100 nm). **c** XRD spectra of the pristine and perovskite films with TTC, C_60_, and TTC/C_60_ layer. **d** UV–Vis spectra of the perovskite films with different passivation layers

Figure 1a shows the device structure used in this study, where the TTC tunneling junction locates at the Ag electrode side. The cross-sectional image analyzed by FE-SEM is displayed in Fig. 1b, in which all functional layers can be clearly distinguished. Hybrid perovskite was deposited using the anti-solvent method. PTAA doped by 1.0 wt% F_4_-TCNQ was used as a hole-transporting layer because the non-wetting surface enables the perovskite to be large grain size [30]. The perovskite precursor solution, which contained 159 mg of CH_3_NH_3_I and 462 mg of PbI_2_ in anhydrous DMF/DMSO (600 μL/78 μL) solution, was then grown on PTAA [31]. TTC is inserted between the perovskite and the C_60_ electron collecting layer as the passivation material. The doped PTAA and the perovskite were deposited by spin coating, while the TTC and C_60_ layer was evaporated along with the top contact consisting of 6 nm BCP and 100 nm Ag. It is noteworthy that no additional process was conducted for the TTC layer.

We carried out XRD of the four perovskite/organic combinations (Fig. 1c), including the pristine perovskite, perovskite/TTC, perovskite/C_60_, and perovskite/TTC/C_60_, to show the phase of TTC on the surface. We found that the position of the diffraction peaks for the perovskite with TTC, C_60_, and TTC/C_60_ is almost as same as the pristine perovskite. The similar half-widths of all the perovskites suggest that organic combinations have little impact on the crystal size. Interestingly, we observed a significant decrease in the intensity of the diffraction peak for TTC/C_60_-coated perovskite, which might be a result of the improved coverage of the perovskite surface [32]. The UV–Vis spectra of these perovskite films (Fig. 1d) were collected to explore the role of TTC on light absorption. Compared to the pristine perovskite, we observed a similar absorbance behavior of the films containing the various passivation layers. The thickness of the normal perovskite film is 450 nm, as estimated from the cross-sectional SEM (Fig. 1b). The passivation layer with a negligible thickness affords a very little effect on light harvesting.

**Fig. 2:**
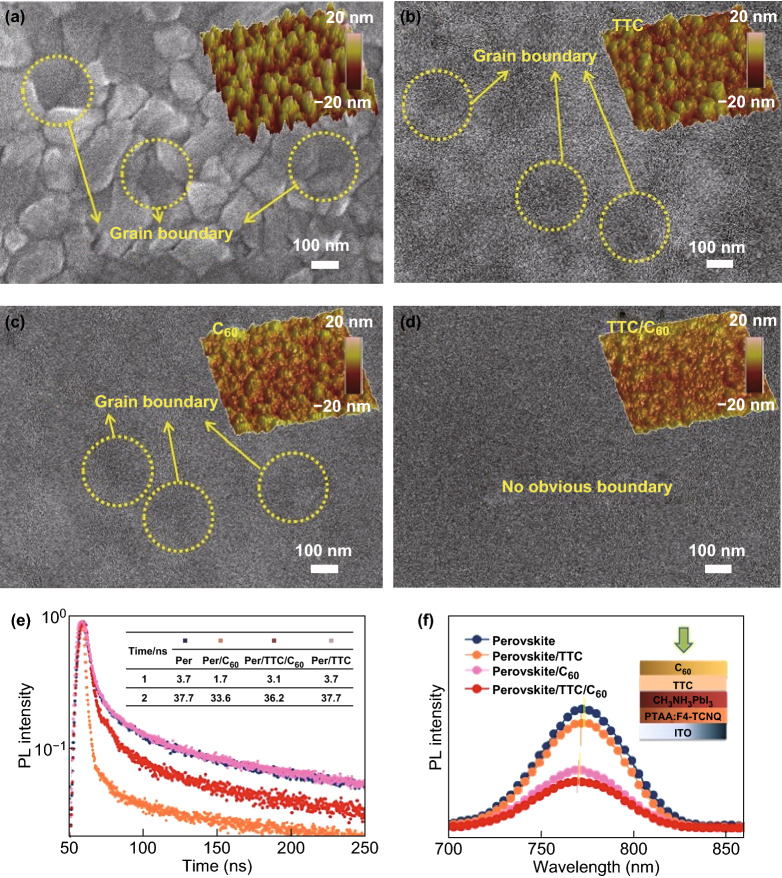
Top-view SEM and AFM images of **a** the normal perovskite and the perovskite with **b** TTC, **c** C_60_, and **d** TTC/C_60_ (scale bar: 100 nm). **e** TRPL and **f** PL spectra of corresponding films on ITO substrate

We infer that the TTC coating fills the vacancies on the surface and the grain boundary of the perovskite, resulting in the passivation effect on the surface defects and decreased charge recombination. To verify this hypothesis, we recorded the top-view SEM images and atomic force microscopy (AFM) images of the normal, TTC, C_60_, and TTC/C_60_ coated perovskite. From the SEM results (Fig. 2), it is observed that both C_60_ and TTC can be deposited at the grain surface/boundary and fill the defects of the films. The decreased root mean square (RMS) from 6.57 nm (perovskite) to 6.45 (TTC/perovskite), 3.03 (C_60_/perovskite), and 2.60 nm (C_60_/TTC/perovskite) indicates the reduced the surface roughness and improved coverage of the perovskite surface, which agrees well with the XRD results. Moreover, the AFM image of the perovskite/TTC/C_60_ film shows a more uniform and flat surface, indicating that TTC/C_60_ is deposited at the grain boundary and reduces the height difference between the grain surface and grain boundary [23].

Furthermore, we evaluated the quality of these films by surveying the charge dynamics using time-resolved photoluminescence (TRPL). Figure 2e shows representative TRPL traces of the perovskite films without and with the respective passivation layer. The pristine perovskite exhibits a long fluorescent lifetime and a long-lived tail with a fast-decay lifetime (*τ*_1_) of 3.7 ns and slow-decay lifetime (*τ*_2_) of 37.7 ns. The long lifetime reflects the high quality of the perovskite film. The TRPL lifetime vastly reduces after capping the top surface of the perovskite with C_60_ (*τ*_1_ of 1.7 ns, *τ*_2_ of 33.6 ns), which is attributed to the fast electron transfer from the perovskite to the fullerene layer because of the matched energy offset [33]. However, the TRPL lifetime of the TTC-coated perovskite is almost the same as the original perovskite, which indicates that electrons cannot transfer from the perovskite to TTC because of the insulativity of TTC. (TTC-only has no effect.) Interestingly, inserting the TTC interlayer into the perovskite and C_60_ increases the PL lifetimes. The perovskite/TTC/C_60_ films decay to background level on timescales longer (*τ*_1_ of 3.1 ns, *τ*_2_ of 36.2 ns) than those of the perovskite/C_60_ samples, although still faster than the pristine perovskite films. To understand the reason for the increased PL lifetimes, we further measured the steady-state PL of all samples. As shown in Fig. 2f, the pristine perovskite shows a strong PL intensity with a peak centered at 774 nm. The perovskite passivated by C_60_ has a decreased PL intensity with a slightly blueshifted peak to 771 nm, which is attributed to the passivated trap states on the surface and/or along the grain boundaries of the perovskite. As expected, the steady-state PL of the TTC passivation sample exhibits a weaker PL intensity. The result shows that the alkyl chain axes of TTC can fill the interface states such as defect states or metal-induced gap states and passivate perovskite film [34].

**Fig. 3:**
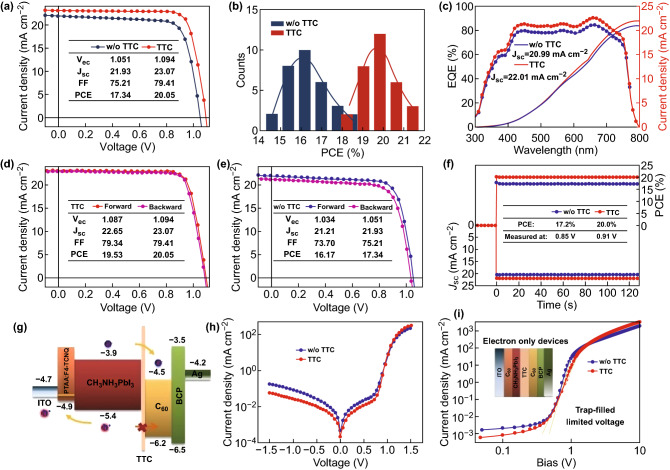
**a** *J*–*V* characteristics of the devices without and with TTC. **b** Statistics of PCE distribution for devices without and with TTC (a batch of 32 solar cells). **c** EQE and integrated current curves of devices without and with TTC. Current–voltage hysteresis curves of the devices **d** with TTC layers and **e** without TTC layers. **f** Steady-state measurement of *J*_sc_ and PCE for the devices without and with TTC layers. **g** Energy band level diagram of the corresponding materials used in PVSCs. **h** *J*–*V* characteristics of devices without and with TTC in dark. **i** *J*–*V* characteristics of electron-only devices without and with TTC. The structures of electron-only device are inserted

In the following discussion, we compared the photovoltaic performance of the perovskite devices without and with different thicknesses of TTC layers. All the fabrication and measurement processes were conducted in dehumidified atmosphere. The photovoltaic parameters were collected under simulated solar illumination (AM 1.5, 100 mW cm^−2^) and are listed in Table 1. The control device based on C_60_ delivered an open-circuit voltage (*V*_oc_) of 1.051 V, a short-circuit current density (*J*_sc_) of 21.93 mA cm^−2^, a FF of 75.21%, and a PCE of 17.34%, which represent a typical performance of fullerene passivated devices. The performance of the device with TTC thickness of 0.5 nm resembles the control device, which might be ascribed to the fact that the TTC film is barely continuous. When the TTC thickness was increased beyond 1 nm, the device performance was significantly increased. The best devices were made with a TTC thickness of 2 nm. The device exhibits a slightly higher *V*_oc_ of 1.084 V, a higher *J*_sc_ of 23.07 mA cm^−2^, and an increased FF of 79.41%, yielding a champion PCE of 20.05%. The device performance was severely reduced when the TTC thickness was increased beyond 3 nm because the electrons failed to tunnel. For comparison, the current density–voltage (*J*–*V*) curves of the control and optimized TTC-inserted devices are shown in Fig. 3a, and the efficiency histogram was summarized from a batch of 32 devices for each type. As shown in Fig. 3b, both types showed relatively narrow PCE distribution, and the majority lied between 15.5–17.5 and 18.5–20.5%, respectively. Figure 3c displays the external quantum efficiency (EQE) responses of the control and device with TTC layer. An enhanced response appeared for the TTC-modified devices at a wavelength range of 350–800 nm. The calculated current densities from the integration of the EQE spectra are 20.99 and 22.01 mA cm^−2^, respectively. These results agree well with the values obtained from the solar simulator [35].

**Table 1:** Average performance parameters of perovskite devices with different thicknesses of TTC tunneling layers

TTC (nm)	*V*_oc_ (V)	*J*_sc_ (mA cm^−2^)	FF (%)	PCE (%)
0	1.051	21.933	75.21	17.336
0.5	1.052	22.031	76.37	17.707
1	1.069	22.274	77.33	18.419
2	1.094	23.075	79.41	20.053
3	1.040	20.698	74.82	16.104

It is also worth mentioning that the devices with TTC exhibit a negligible hysteresis compared to the C_60_-only devices, which suggests that the TTC passivation layer can block the ion migration channel at the grain boundary; moreover, ion migration at grain boundaries plays a dominant role in the photocurrent hysteresis. The absence of photocurrent hysteresis is confirmed by altering the photocurrent scanning direction (Fig. 3d, e). Besides, we also performed the steady-state photocurrent measurement at the maximum power output point. The control device shows a photocurrent of 20.3 mA cm^−2^ and a PCE of 17.2%. The modified device indicates a photocurrent of 22.0 mA cm^−2^ and a PCE of 20.0% (Fig. 3f), which is consistent with the *J*–*V* results [36].

We also illustrated the function of TTC passivation layer on transporting electrons and blocking holes using the proposed energy diagram in Fig. 3g. The C–C–C plane of TTC can form a typical organic ultrathin insulator film, which would adjust the band alignment of the devices. We measured the conductivity of the ITO/PTAA:F_4_-TCNQ/TTC/C_60_/BCP/Ag and ITO/PTAA:F_4_-TCNQ/C_60_/BCP/Ag films via the current–voltage (*I*–*V*) curve measurement and show this in Fig. S1. It can be seen that their conductivity has little variation, suggesting that TTC has little impact on the electron transport inside the devices because of the small thickness of TTC. The photogenerated electrons at the conduction band of the perovskite can tunnel into the lowest unoccupied molecular orbital (LUMO) of C_60_, because there are energy-matching unoccupied states in C_60_ for electrons to tunnel into. On the contrary, the tunneling rate of the holes will be very low because there are no unoccupied states for the holes to tunnel into. Therefore, the TTC layer allows electron transfer from the perovskite to C_60_ layer by tunneling and blocks the holes, which reduces their recombination at the interface [37]. The dark current density of the TTC-inserted device was suppressed at negative bias (Fig. 3h), suggesting a reduced leakage current density and an increased shunt resistance. To further prove the effect of the electron injection efficiency at the perovskite/TTC/C_60_ interface, we fabricated electron-only devices with the configuration of ITO/C_60_/perovskite TTC/C_60_/Ag and ITO/C_60_/perovskite/C_60_/Ag. As shown in Fig. 3i, the TTC-modified device exhibits a smaller trap-filled limited voltage, implying that lower electron trap density occurs at the perovskite/TTC/C_60_ interface [38–40].

**Fig. 4:**
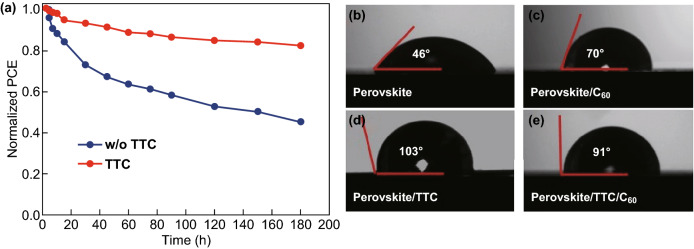
**a** Device stability of control and TTC-based PVSCs. The contact angles of different films: **b** perovskite, **c** perovskite/C_60_, **d** perovskite/TTC, and **e** perovskite/TTC/C_60_

The hydrophobic small molecule on the perovskite films could form a water-resistant layer to protect the perovskite film from water damage. In this regard, we tested the shelf stability of corresponding PVSCs at an ambient environment of 40% relative humidity in air without encapsulation. As shown in Fig. 4a, it is found that the PVSCs with TTC exhibited a better long-term stability compared with the standard C_60_-based devices. To obtain more direct evidence of the device stability, we further investigated the contact angle of the pristine C_60-_, TTC-, and C_60_/TTC-containing perovskite films. The significant variation of device stability resulted from different hydrophobicities of the passivation layer (Fig. 4b–e). The TTC film shows a very big water contact angle of 103°, so that the hydrophobic TTC layer can efficiently prevent the water penetration into the perovskite. The angle reduces slightly to 91° after the coating of C_60_ on TTC, which is still much bigger than that of C_60_-only films. The improved coverage and hydrophobicity of the perovskite layer protect the PVSCs from air and water, which slows the CH_3_NH_3_PbI_3_ decomposition [41–43]. We also measured the evolution of PCE over time under continuous illumination for the non-encapsulated devices without and with TTC. As shown in Fig. S2, both solar cells decay to about 40% of their initial efficiency within 5 h when subjected to full spectrum sunlight, which illustrates that TTC steadily works inside the device.

## Conclusion

In summary, a hydrophobic small molecular TTC was demonstrated as an effective interlayer for planar p-i-n PVSCs. The insertion of TTC reduces the interface trap density and enhances the electron extraction at the perovskite/C_60_ interface. With the device structure of ITO/PTAA:F_4_TCNQ/CH_3_NH_3_PbI_3_/TTC/C_60_/BCP/Ag, we achieved a PCE of 20.05% with a high FF of 79.41%. Moreover, the hydrophobic TTC successfully protected the perovskite film from water damage as a water-resistant layer on the perovskite films. Thus, PVSCs with better long-term operation stability were realized. This study provides an efficient method using small molecular to improve the efficiency and stability of the PVSCs.

## Electronic supplementary material

Below is the link to the electronic supplementary material.

Supplementary material 1 (PDF 252 kb)

## References

[1] Kojima A, Teshima K, Shirai Y, Miyasaka T (2009). Organometal halide perovskites as visible-light sensitizers for photovoltaic cells. J. Am. Chem. Soc..

[2] Sha WE, Ren X, Chen L, Choy WC (2015). The efficiency limit of CH_3_NH_3_PbI_3_ perovskite solar cells. Appl. Phys. Lett..

[3] Xiao Y, Cheng N, Kondamareddy KK, Wang C, Liu P, Guo S, Zhao XZ (2017). W-doped TiO_2_ mesoporous electron transport layer for efficient hole transport material free perovskite solar cells employing carbon counter electrodes. J. Power Sources.

[4] Li Z, Liu C, Zhang X, Ren G, Han W, Guo W (2019). Developing 1D Sb-embedded carbon nanorods to improve efficiency and stability of inverted planar perovskite solar Cells. Small.

[5] Song Z, McElvany CL, Phillips AB, Celik I, Krantz PW (2017). A technoeconomic analysis of perovskite solar module manufacturing with low-cost materials and techniques. Energy Environ. Sci..

[6] Li Z, Zhao Y, Wang X, Sun Y, Zhao Z, Li Y, Zhou H, Chen Q (2018). Cost analysis of perovskite tandem photovoltaics. Joule.

[7] Xiao Y, Wang C, Kondamareddy KK, Liu P, Qi F, Zhang H, Guo S, Zhao XZ (2019). Enhancing the performance of hole-conductor free carbon-based perovskite solar cells through rutile-phase passivation of anatase TiO_2_ scaffold. J. Power Sources.

[8] *Best Research*-*Cell Efficiency Chart.* NREL chart. https://www.nrel.gov/pv/cell-efficiency.html. Accessed 15 May 2019

[9] Leijtens T, Eperon GE, Noel NK, Habisreutinger SN, Petrozza A, Snaith HJ (2015). Stability of metal halide perovskite solar cells. Adv. Energy Mater..

[10] Niu G, Li W, Meng F, Wang L, Dong H, Qiu Y (2014). Study on the Stability of CH_3_NH_3_PbI_3_ films and the effect of post-modification by aluminum oxide in all-solid-state hybrid solar cells. J. Mater. Chem. A.

[11] Leguy AM, Hu Y, Campoy-Quiles M, Alonso MI, Weber OJ (2015). Reversible hydration of CH_3_NH_3_PbI_3_ in films, single crystals, and solar cells. Chem. Mater..

[12] Song Z, Abate A, Watthage SC, Liyanage GK, Phillips AB, Steiner U, Graetzel M, Heben MJ (2016). Perovskite solar cell stability in humid air: partially reversible phase transitions in the PbI_2_-CH_3_NH_3_I-H_2_O system. Adv. Energy Mater..

[13] Müller C, Glaser T, Plogmeyer M, Sendner M, Döring S (2015). Water infiltration in methylammonium lead iodide perovskite: fast and inconspicuous. Chem. Mater..

[14] Saidaminov MI, Kim J, Jain A, Quintero-Bermudez R, Tan H (2018). Suppression of atomic vacancies via incorporation of isovalent small ions to increase the stability of halide perovskite solar cells in ambient air. Nat. Energy.

[15] Zheng H, Liu G, Zhu L, Ye J, Zhang X (2018). The effect of hydrophobicity of ammonium salts on stability of quasi-2D perovskite materials in moist condition. Adv. Energy Mater..

[16] Zhu Z, Chueh CC, Lin F, Jen AK (2016). Enhanced ambient stability of efficient perovskite solar cells by employing a modified fullerene cathode interlayer. Adv. Sci..

[17] Li Z, Liu C, Zhang X, Guo J, Cui H, Shen L, Bi Y, Guo W (2019). Using easily prepared carbon nanodots to improve hole transport capacity of perovskite solar cells. Mater. Today Energy.

[18] Hwang I, Jeong I, Lee J, Ko MJ, Yong K (2015). Enhancing stability of perovskite solar cells to moisture by the facile hydrophobic passivation. ACS Appl. Mater. Interfaces.

[19] Zhao Y, Zhang H, Ren X, Zhu HL, Huang Z (2018). Thick TiO_2_-based top electron transport layer on perovskite for highly efficient and stable solar cells. ACS Energy Lett..

[20] Zong Y, Zhou Y, Zhang Y, Li Z, Zhang L (2018). Continuous grain-boundary functionalization for high-efficiency perovskite solar cells with exceptional stability. Chem.

[21] Li X, Dar MI, Yi C, Luo J, Tschumi M, Zakeeruddin SM, Nazeeruddin MK, Han H, Grätzel M (2015). Improved performance and stability of perovskite solar cells by crystal crosslinking with alkylphosphonic acid ω-ammonium chlorides. Nat. Chem..

[22] Yu JC, Badgujar S, Jung ED, Singh VK, Kim DW (2019). Highly efficient and stable inverted perovskite solar cell obtained via treatment by semiconducting chemical additive. Adv. Mater..

[23] Wang C, Song Z, Zhao D, Awni RA, Li C (2019). Improving performance and stability of planar perovskite solar cells through grain boundary passivation with block copolymer. Solar RRL.

[24] Luo H, Lin X, Hou X, Pan L, Huang S, Chen X (2017). Efficient and air-stable planar perovskite solar cells formed on graphene-oxide-modified PEDOT: PSS hole transport layer. Nano-Micro Lett..

[25] Vidyasagar CC, Flores BMM, Pérez VMJ (2018). Recent advances in synthesis and properties of hybrid halide perovskites for photovoltaics. Nano-Micro Lett..

[26] Liu Z, Sun B, Liu X, Han J, Ye H, Shi T, Tang Z, Liao G (2018). Efficient carbon-based CsPbBr 3 inorganic perovskite solar cells by using Cu-phthalocyanine as hole transport material. Nano-Micro Lett..

[27] Petrus ML, Schutt K, Sirtl MT, Hutter EM, Closs AC (2018). New generation hole transporting materials for perovskite solar cells: amide-based small-molecules with nonconjugated backbones. Adv. Energy Mater..

[28] Jung SK, Heo JH, Lee DW, Lee SC, Lee SH (2018). Nonfullerene electron transporting material based on naphthalene diimide small molecule for highly stable perovskite solar cells with efficiency exceeding 20%. Adv. Funct. Mater..

[29] Deng W, Liang X, Kubiak PS, Cameron PJ (2018). Molecular interlayers in hybrid perovskite solar cells. Adv. Energy Mater..

[30] Wolff CM, Zu F, Paulke A, Toro LP, Koch N, Neher D (2017). Reduced interface-mediated recombination for high open-circuit voltages in CH_3_NH_3_PbI_3_ solar cells. Adv. Mater..

[31] Elseman AM, Sharmoukh W, Sajid S, Cui P, Ji J (2018). Superior stability and efficiency over 20% perovskite solar cells achieved by a novel molecularly engineered rutin-AgNPs/thiophene copolymer. Adv. Sci..

[32] Xiao Y, Wang C, Kondamareddy KK, Cheng N, Liu P (2018). Efficient electron transport scaffold made up of submicron TiO_2_ spheres for high-performance hole-transport material free perovskite solar cells. ACS Appl. Energy Mater..

[33] Choi H, Mai CK, Kim HB, Jeong J, Song S (2015). Conjugated polyelectrolyte hole transport layer for inverted-type perovskite solar cells. Nat. Commun..

[34] Shao Y, Xiao Z, Bi C, Yuan Y, Huang J (2014). Origin and elimination of photocurrent hysteresis by fullerene passivation in CH_3_NH_3_PbI_3_ planar heterojunction solar cells. Nat. Commun..

[35] Hsu HL, Hsiao HT, Juang TY, Jiang BH, Chen SC, Jeng RJ, Chen CP (2018). Carbon nanodot additives realize high-performance air-stable p-i-n perovskite solar cells providing efficiencies of up to 20.2%. Adv. Energy Mater..

[36] Zhang F, Song J, Hu R, Xiang Y, He J (2018). Interfacial passivation of the p-doped hole-transporting layer using general insulating polymers for high-performance inverted perovskite solar cells. Small.

[37] Ren X, Wang Z, Sha WE, Choy WC (2017). Exploring the way to approach the efficiency limit of perovskite solar cells by drift-diffusion model. ACS Photon..

[38] Fang HH, Wang F, Adjokatse S, Zhao N, Even J, Loi MA (2016). Photoexcitation dynamics in solution-processed formamidinium lead iodide perovskite thin films for solar cell applications. Light: Sci. Appl..

[39] Xie C, You P, Liu ZK, Li L, Yan F (2017). Ultrasensitive broadband phototransistors based on perovskite/organic-semiconductor vertical heterojunctions. Light: Sci. Appl..

[40] Wu T, Wang Y, Li X, Wu Y, Meng X, Cui D, Yang X, Han L (2019). Efficient defect passivation for perovskite solar cells by controlling the electron density distribution of donor-π-acceptor molecules. Adv. Energy Mater..

[41] Gu LL, Fan ZY (2017). Perovskite/organic-semiconductor heterojunctions for ultrasensitive photodetection. Light: Sci. Appl..

[42] Yu JC, Badgujar S, Jung ED, Singh VK, Kim DW (2019). Highly efficient and stable inverted perovskite solar cell obtained via treatment by semiconducting chemical additive. Adv. Mater..

[43] Zhao YC, Zhou WK, Zhou X, Liu KH, Yu DP, Zhao Q (2017). Quantification of light-enhanced ionic transport in lead iodide perovskite thin films and its solar cell applications. Light: Sci. Appl..

