# Supplementary material for: Surface Passivation of Perovskite Solar Cells Toward Improved Efficiency and Stability
Source: Nanomicro Lett. 2019 Jun 7;11:50. doi: 10.1007/s40820-019-0282-0 (PMC7770653; doi:10.1007/s40820-019-0282-0)
Supplement: Supplementary file 1 — Supplementary material 1 (PDF 252 kb) [file 40820_2019_282_MOESM1_ESM.pdf]

Supporting Information for

Surface Passivation of Perovskite Solar Cells toward Improved Efficiency and Stability

Zhiqi Li[1], Jiajun Dong[2], Chunyu Liu[1], Jiaxin Guo[1], Liang Shen[1], Wenbin Guo [1, *]

[1]State Key Laboratory on Integrated Optoelectronics, College of Electronic Science and Engineering , Jilin University, 2699 Qianjin Street, Changchun 130012, People's Republic of China

[2]State Key Laboratory on Superhard Materials, Jilin University, 2699 Qianjin Street, Changchun 130012, People's Republic of China

Zhiqi Li and Jiajun Dong contributed equally to this work

*Corresponding author. E-mail: guowb@jlu.edu.cn (W.B. Guo)

Supplementary Table and Figures

Table S1 Photovoltaic Parameters of the Interfacial Engineering in Previous Reports

Time	Structure	V_{oc}	J_{sc}	FF	PCE	References
2015	ITO/TiO_2/cross-linked $MAPbI_3$/Spior-OMeTAD/Au	1.00	22.05	0.75	16.55	Nat. Chem. 7,703 (2015)
2015	ITO/PTAA/$MAPbI_3$/PCBM/BCP/AL	1.07	22.00	76.8	18.1	Nat. Commun. 6, 7747 (2015)
2016	ITO/PTAA/$MAPbI_3$/PS/C_{60}/BCP/Ag	1.10	22.90	77.6	20.3	Adv. Mater. 28, 6734 (2016)
2017	ITO/TiO_2/$MAPbI_3$/ZrO_2/C	1.054	21.45	70.3	15.95	Nat. Commun. 8, 15684 (2017)
2017	ITO/PEDOT:PSS:GO/$MAPbI_3$/PCBM/Ag	0.94	21.92	74.78	15.34	Nano-Micro Lett. 9, 39 (2017)
2018	ITO/TiO_2-Cl/CsMAFA/Spior-OMeTAD/Au	1.196	22.2	76.4	20.3	Science 355, 722(2017).
2018	ITO/NiO/$MAPbI_3$/TiO2/Ag	1.14	21.96	82.00	20.50	ACS Energy Lett. 3, 2891 (2018)
2018	ITO/PTAA/PS/$MAPbI_3$/PCBM/Bphen/Ag	1.12	23.38	77.10	20.16	Small 14, 1704007 (2018)
2018	ITO/NiO/$MAPbI_3$/PCBM/PPDIN6/Ag	1.08	22.68	83.40	20.43	Nano Energy 52, 300 (2018)
2018	ITO/TiO_2/$MAPbI_3$/PHPT-Py/Rutin-AgNP/Ag	1.11	25.25	74.05	20.78	Adv. Sci. 1800568 (2018)
2018	ITO/TiO_2-Cl/CsMAFA/Spior-OMeTAD/Au	1.15	23.80	0.77	21.10	Adv. Funct. Mater. 28, 1706287 (2018)
2019	ITO/TiO_2/$MAPbI_3$/C	0.97	21.47	67.04	13.98	J. Power Sources 422, 138 (2019)
2019	ITO/TiO_2-Cl/CsMAFA/Spior-OMeTAD/Au	1.19	23.2	78.2	20.49	Adv. Energy Mater. 9, 1802646 (2019)
2019	ITO/A14083/$MAPbI_3$/PCBM/Bphen/Ag	1.13	22.4	80.00	20.30	Adv. Mater. 31, 1805554 (2019)

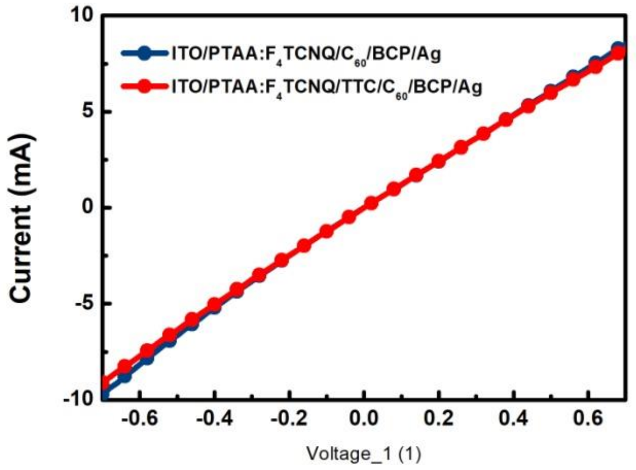

Fig. S1 Current-voltage (*I-V*) curves of the ITO/PTAA:F_4TCNQ/TTC/C_{60}/BCP/Ag and ITO/PTAA:F_4TCNQ/C_{60}/BCP/Ag films

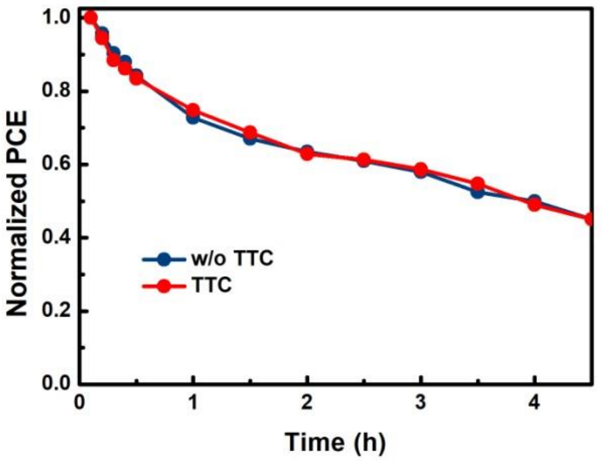

Fig. S2 Evolution of PCE for normalized solar cell without and with TTC under continuous illumination
